# Evaluation of a novel autoinjector for subcutaneous self-administration of belimumab in systemic lupus erythematosus 

**DOI:** 10.5414/CP202623

**Published:** 2016-09-26

**Authors:** Saira Z. Sheikh, Anne E. Hammer, Norma Lynn Fox, James Groark, Herbert Struemper, David Roth, David Gordon

**Affiliations:** 1 University of North Carolina, Chapel Hill,; 2 GSK, Research Triangle Park, NC,; 3 GSK, Potomak, MD,; 4 GSK, Philadelphia, PA, and; 5 PAREXEL International, Research Triangle Park, NC, USA

**Keywords:** belimumab, autoinjector, usability, reliability, pharmacokinetics

## Abstract

Objective: To study self-administration and pharmacokinetics (PK) of subcutaneous (SC) belimumab in patients with systemic lupus erythematosus (SLE). Methods: Patients previously treated with belimumab self-administered belimumab 200 mg SC weekly for 8 weeks using an autoinjector. The primary endpoint was the proportion of patients able to self-administer their first and second dose (weeks 1 and 2) in the clinic. The proportion able to self-administer at weeks 4 and 8 (clinic) and weeks 3, 5, 6, and 7 (home) were secondary endpoints. Belimumab PK, safety, and injection-site pain were assessed. Results: 91/95 patients completed the study (withdrawals: adverse events (AEs): 3; lost to follow-up: 1). 93% were female, and mean (SD) age was 44.8 (12.50) years. The majority (99%, 89/90; no attempt, n = 5) successfully self-administered belimumab SC at weeks 1 and 2 (5 had clinic staff assistance), and 98% (85/87) successfully self-administered at weeks 4 and 8. Home-administration success rates were high (93%, (81/87) at weeks 3, 5, 6, and 7). Week 8 median trough concentration was 113 µg/mL. For patients with a ≤ 1.5-week interval between IV SC administration, week-1 concentrations were higher vs. week 8 (+ 51% median) but within a range observed with IV dosing; those with a ≥ 2.5-week interval had median differences close to 0. AEs and serious AEs were low, with no deaths; pain levels were low and decreased with subsequent injections. Conclusion: Patients with SLE successfully self-administered belimumab SC using a novel autoinjector; the PK profile was stable following a switch from IV with acceptable AE and pain levels. The recommended dosing interval between IV to SC dosing is 1 – 4 weeks.

## Introduction 

Systemic lupus erythematosus (SLE) is a chronic, autoimmune, inflammatory disease that causes considerable burden to patients and society [1]. Belimumab is a human, monoclonal antibody that binds to and inhibits the biological activity of B-lymphocyte stimulator (BLyS) [2]. The efficacy and safety of intravenous (IV) belimumab were demonstrated in two large, randomized, multicenter, placebo-controlled trials, BLISS 52, and BLISS 76 [3, 4]. Subsequently, belimumab 10 mg/kg IV was approved for use in adults with active, autoantibody-positive SLE, as an add-on to standard SLE therapy [5]. The dosing regimen for belimumab IV requires administration in an infusion center every 4 weeks. Devices for subcutaneous (SC) delivery of belimumab, which are safe and easy to self-administer, would be a significant advance for patients with active disease. 

Several SC treatments are available to treat rheumatoid arthritis (RA) [6, 7, 8, 9]. SC treatment enabled patients with RA to continue their everyday routine and reduced the need to travel to an infusion center, a factor that resulted in more patients choosing a SC treatment [10]. There is a high level of patient acceptance with autoinjector devices in RA, including patients with severe hand disability [11]. Musculoskeletal and mucocutaneous manifestations, such as fatigue, joint pain, and swelling, are common in patients with active SLE, particularly in the fingers, hands, and wrists, and can impact the patient’s ability to carry out day-to-day tasks [12, 13, 14]. As patients with SLE have similar therapeutic needs to patients with RA, autoinjector usability should be studied in these patients. A novel single-use autoinjector has been developed for SC delivery of belimumab. In a single-dose study of healthy volunteers, the autoinjector demonstrated good usability, reliability, and safety following self-administration [15]. Four weekly doses of belimumab 200 mg SC were well tolerated in healthy volunteers, and it was predicted that weekly administration would achieve exposures comparable to the approved IV dosing regimen [16]. As patients currently treated with belimumab IV may choose to switch to the SC formulation following regulatory approval, the pharmacokinetic (PK) profile across the IV-to-SC switch should be examined to determine the optimum dosing interval. 

The aims of this study were to assess the suitability of a novel autoinjector for self-administration of belimumab by patients with active SLE under real-life conditions, change in PK trough concentrations when switching from IV to SC administration, and safety and tolerability of belimumab administered via the autoinjector. 

## Methods 

### Study design and treatment 

**Figure 1.**
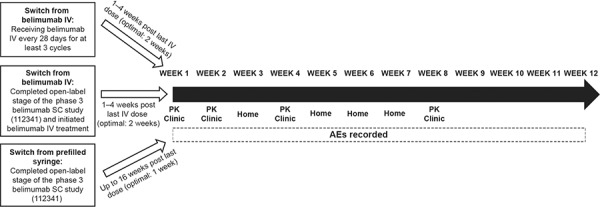
Study design. AE = adverse events; clinic = self-administration of belimumab SC at the clinic; home = self-administration of belimumab SC at home; IV = intravenous; PK = blood sample taken for pharmacokinetic analysis prior to belimumab administration; SC = subcutaneous.

This was an open-label, single-arm, multi-dose study in patients with SLE (GSK protocol 200339; NCT02124798). The study was conducted in accordance with the Declaration of Helsinki 2008 [17] and approved by an Institutional Review Board. All patients provided written, informed consent prior to study enrollment. Adult patients (> 18 years) with active, autoantibody-positive SLE (American College of Rheumatology criteria [18]) receiving belimumab treatment were included. Patients were eligible to switch from belimumab IV or belimumab SC (prefilled syringe) to belimumab SC (autoinjector) and could continue their other SLE treatments (Figure 1). Patients who switched from belimumab IV to belimumab SC (autoinjector) must have received belimumab IV every 4 weeks for at least three 4-week cycles or have completed the open-label phase of a phase-3 belimumab SC study (prefilled syringe) (GSK protocol BEL112341; NCT01484496) and initiated treatment with belimumab IV. Day 0 (first dose belimumab SC) was targeted to be 1 – 4 weeks (~ 2 weeks) after the last dose of belimumab IV. Patients who completed the prior belimumab SC study, but who did not then initiate treatment with belimumab IV, could also participate. For this switch from belimumab SC (prefilled syringe) to belimumab SC (autoinjector), the first dose administered using the autoinjector was scheduled up to 16 weeks (target 1 week) after the last dose of belimumab SC by prefilled syringe. 

**Figure 2.**
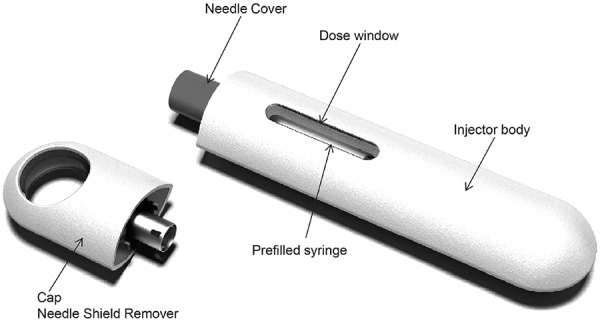
Autoinjector device. Registered design protection granted/pending. Image originally published in Struemper et al. 2015 [15].

Patients self-administered belimumab 200 mg SC (1 mL) using the autoinjector device (Figure 2) for 8 weekly doses, alternating between thigh and abdomen injection sites. Patients received training on the use of the autoinjector at screening and prior to their first injection (week 1). Training consisted of reviewing the instructions for use and performing a supervised practice injection using a foam pad. Injections at weeks 1, 2, 4, and 8 were performed in the clinic, and injections at weeks 3, 5, 6, and 7 were performed outside the clinic (home). Patients were supervised during the first injection and could receive support from site staff at all visits. Patients attended an exit visit 4 weeks after the final injection. At study completion, patients could resume or initiate treatment with belimumab IV (10 mg/kg every 4 weeks) 2 – 3 weeks after the last belimumab SC dose; IV loading doses were not required. The recommended time intervals between last IV dose and the first SC dose, and between last SC dose and restarting IV administration, were chosen such that the new SC or resumed IV regimens started close to their predicted steady-state trough concentrations [16]. 

### Endpoints and assessments 

The primary endpoint was the proportion of patients successfully able to self-administer their first and second doses (weeks 1 and 2) in the clinic, monitored by site staff. The proportion of patients successfully able to self-administer doses in weeks 4 and 8 (clinic) and the proportion of patients who recorded successful self-administration at home in weeks 3, 5, 6, and 7 were secondary endpoints. In order to assess usability and reliability, the rate of successful (fully complete) self-administered injections relative to attempted self-administered injections was assessed. Patients used diaries to record injection details (e.g., time, date, injection site), assess the completeness of the injection, and document their experience with the autoinjector. Clinic injections were assessed by site staff. An injection was considered successful if the correct injection site (abdomen or thigh) was used, if the device was correctly actuated while placed on the injection site, and if the device was held in place until the full dose was delivered. Patients were permitted a second attempt; if the second attempt was successful, this was deemed an overall successful injection. Percent compliance was calculated as 100 X ([number of injections prescribed – number of injections missed]/ number of injections prescribed). 

Blood samples for PK analyses were taken at weeks 1, 2, 4, and 8 prior to SC dosing and belimumab trough concentrations (C_min_) were assessed. According to the prespecified analysis plan, the difference between week-8 and week-0 concentrations was analyzed relative to the case report form (CRF)-recorded time interval between the last IV and the first SC dose and according to body mass index (BMI) categories (underweight: < 18.5, normal: 18.5 to < 25, overweight: 25 to < 30 and obese: ≥ 30 kg/m^2^). Several patients received their first SC dose more than the targeted 4 weeks after their last IV dose; these patients were not excluded from the analysis. After database lock and generation of Figure 6 and the associated statistical summary, 3 of the 95 patients in the PK population were determined to have shorter time intervals between the last IV and first SC dose than recorded in the CRF (1 vs. 6 weeks, 7.3 vs. 7.6 weeks, 3 vs. 7 weeks, confirmed by site vs. recorded in the database, respectively). 

Safety was assessed using adverse events (AEs) and serious adverse events (SAEs), recorded from the first injection through week-12 exit visit, and changes in laboratory values. Pain was assessed using a visual analog scale (VAS; 0 – 100 mm) immediately after an injection and 1 and 24 hours post injection, a rating of 0 represented no pain, and 100 worst possible pain. 

### Statistical analysis 

No formal sample size calculation was conducted. The intent-to-treat (ITT) population included all patients who received at least 1 SC dose. For the primary and secondary endpoints, only patients who attempted self-administration of the injection at all relevant time points were included. Patients who had at least 1 post-belimumab serum sample analyzed were included in the PK population. Continuous variables were summarized with the mean, median, standard deviation (SD), 25^th^ and 75^th^ percentiles, minimum and maximum. Categorical variables were summarized with frequency counts and percentages. 

## Results 

### Patient population 

**Figure 3.**
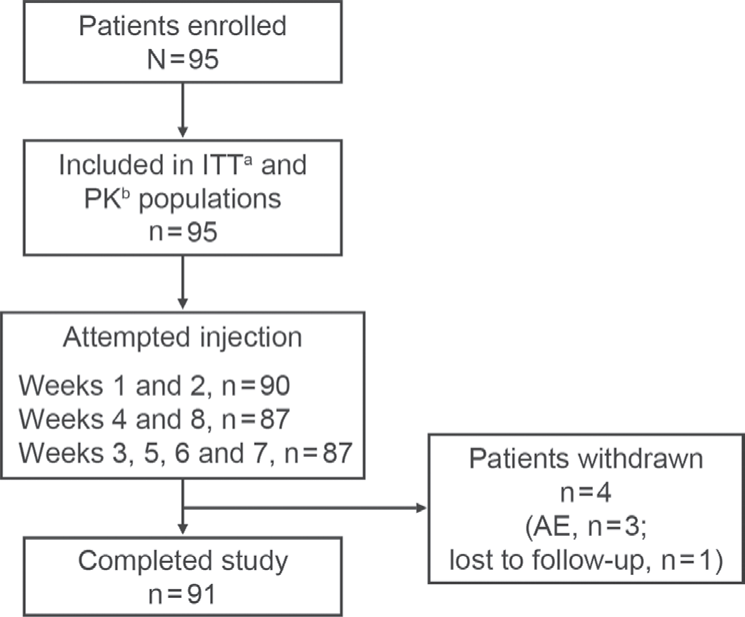
Patient disposition. ^a^Received ≥1 dose belimumab; ^b^patients included in the ITT population for whom ≥1 post belimumab treatment PK sample was analyzed. AE = adverse event; ITT = intent-to-treat; PK = pharmacokinetic.

95 patients enrolled, and 91 completed the study; 3 withdrew due to AEs, and 1 was lost to follow-up (Figure 3). The majority was female (n = 88), white (n = 69), and mean (SD) age was 44.8 (12.50) years (range: 24 – 74 years). Mean (SD) weight was 85.9 (24.70) kg, and mean (SD) BMI was 31.4 (8.68) kg/m^2^. The majority of patients switched directly from belimumab IV (n = 93), and 2 patients switched directly from belimumab SC by prefilled syringe. Following the study, 90 patients resumed treatment with belimumab IV. 

Median exposure was 56 days (range: 14 – 60 days), and 91 patients (96%) attempted 7 or 8 injections. Mean (SD) compliance was 98% (5.15). 

### Successful self-administration 

**Figure 4.**
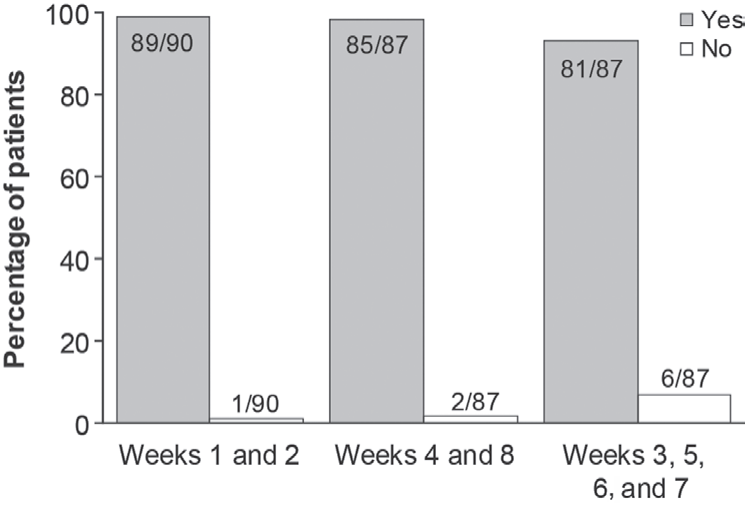
Percentage of successful injections at each week. Data missing for 5 patients at weeks 1 and 2, and for 8 patients at weeks 3 – 8. Data labels represent number of patients.

At weeks 1 and 2, 99% (89/90) of patients who attempted an injection successfully administered belimumab using the autoinjector (Figure 4). At week 2, 5/95 patients (ITT) did not attempt an injection and so were excluded from primary endpoint analysis. At week 1, clinical staff assisted with the injection for 5 patients, and these were included as successful injections. At weeks 4 and 8, 98% (85/87) of patients who attempted an injection successfully self-administered belimumab. Patients reported success in similar proportions at home and in the clinic; at home, 93% (81/87) of patients who attempted an injection successfully self-administered belimumab (weeks 3, 5, 6, and 7). Overall, 5 patients failed a first attempt but completed a second successful injection (1 each at weeks 1, 3, and 5, and 2 at week 7). 

### Pharmacokinetics 

**Figure 5.**
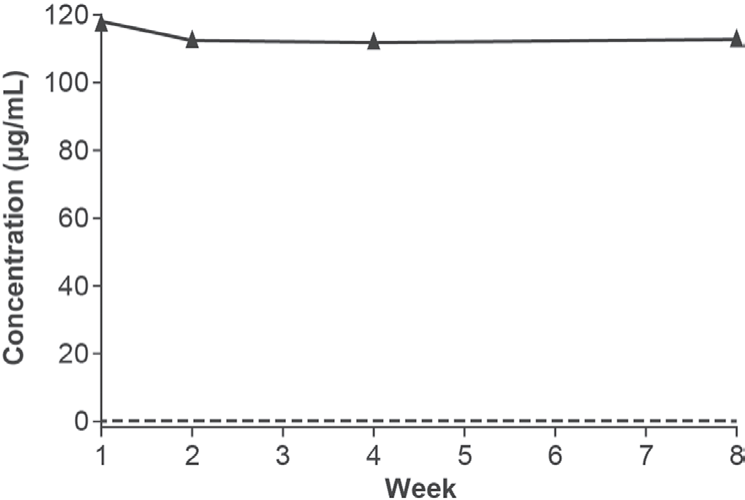
Median belimumab serum concentration by week (PK population). Minimum and maximum values: week 1 (0, 504.29 µg/mL), week 2 (17.47, 467.86 µg/mL), week 4 (34.41, 278.27 µg/mL), week 8 (30.19, 296.47 µg/mL). Dashed line: lower limit of quantification (0.1 µg/mL). PK = pharmacokinetic.

**Figure 6.**
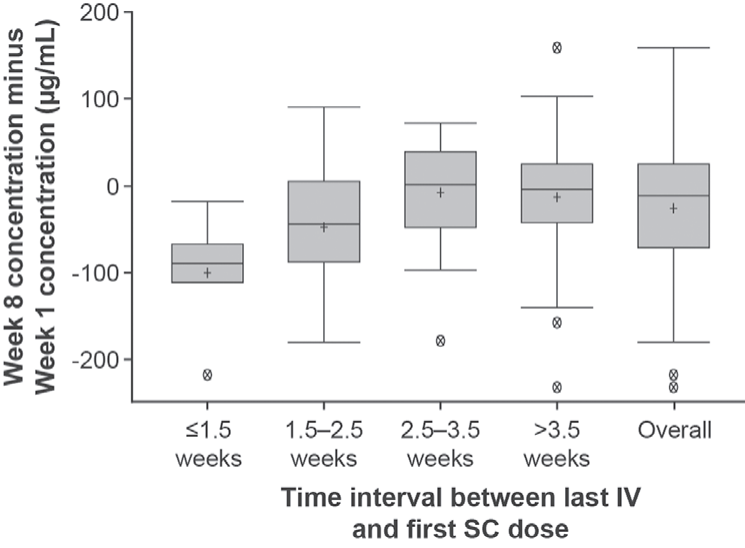
Effect of the IV-to-SC interval on week-8 to week-1 concentration difference (PK population^a^). ^a^Excludes two patients who received belimumab SC via prefilled syringe at study entry. Boxes represent the median, 25^th^ and 75^th^ percentiles. Ends of whiskers represent the maximum observation below the upper fence (75^th^ percentile + 1.5 × interquartile range) and the minimum observation above the lower fence (25^th^ percentile – 1.5 × interquartile range). Crosses (+) represent the mean. Circles with a cross (X) represent outliers. IV = intravenous; PK = pharmacokinetic; SC = subcutaneous.

Median serum belimumab trough concentration at week 8 was 113 µg/mL (range: 30 – 296 µg/mL) (Figure 5). Following the switch from IV to SC, steady-state trough belimumab levels were attained on average by week 2 (Figure 5). When switching from IV to SC administration, median percentage changes in belimumab concentration between week 8 and week 1 were –51%, –23%, 1%, and –3% for patients with intervals of ≤ 1.5 weeks, 1.5 – 2.5 weeks, 2.5 – 3.5 weeks, and > 3.5 weeks, respectively (Figure 6). 

The difference between week 8 and week 1 belimumab concentrations was greater for patients with a normal BMI (BMI 18.5 to < 25; + 14% median) compared with patients classified as overweight (BMI 25 to < 30; + 6% median) and obese (BMI ≥ 30; –34% median). 

### Injection failures 

Overall, 98% (720/736) of attempted injections were successful. Of the unsuccessful injections, 12 were associated with use errors only, 2 were associated with device errors only, and 2 were initially recorded as both a use error and device error, but after follow-up with the clinical site, they were attributed to use errors only (Table 1). Four of the use errors were attributed to one patient and none of the other patients repeated a use error. There were 2 reported device malfunctions, 1 of which was substantiated as an actual device error. 

### Patient diaries 

Across 8 weeks, 4 patients required assistance or further training to complete their injection successfully; this was in addition to the 5 patients who required assistance at week 1, as noted above. The percentage of patients who used the provided instructions generally decreased over time from 94% (89/95 patients) at week 1 to 71% (65/91 patients) at week 8. Use of injection site locations (left thigh, right thigh, left abdomen, right abdomen) was balanced. The majority of patients agreed that the autoinjector was comfortable to hold (≥ 98%) and that injection time was acceptable (≥ 97%). 

### Safety 

Overall, 39 (41%) patients reported at least 1 treatment-emergent AE, and 15 (16%) were considered drug-related (Figure 2). Four (4%) patients reported 7 SAEs; 4 occurred in 1 patient (anemia, neutropenia, pneumonia, pyrexia), and there was 1 case each of postoperative abscess (drug withdrawn), pulmonary embolism, and deep vein thrombosis. Three (3%) patients reported 5 AEs leading to discontinuation (nausea, vomiting, and pyrexia in 1 patient, and 1 case each of herpes zoster and postoperative abscess). Two nonopportunistic infections (herpes zoster) were reported, and 4 postinjection nonserious systemic reactions via broad customized medical dictionary for regulatory activities query (CMQ; cough, pruritus, rash, and swelling). Two severe AEs were reported (1 case each of postoperative abscess and deep vein thrombosis). No deaths occurred.[Table Table2]

Injection site reaction AEs were reported by 4 (4%) patients; none were serious or resulted in study discontinuation. Mean injection site pain (according to the 0 – 100 mm VAS) at dosing was highest with the second injection (8.15 mm) and reduced with repeated administrations (2.30 mm at week 8). Pain levels reduced 1 hour after dosing and ranged from 0.12 to 1.08 (Figure 3). Most patients rated their injection pain as acceptable. Sharp stinging pain was reported most frequently, followed by burning pain (data not shown).[Table Table3]

## Discussion 

This was the first study conducted in patients with SLE to assess use of a novel autoinjector for self-administration of belimumab SC. PK of the switch from belimumab IV to belimumab SC were examined. 

The majority of patients was able to successfully self-administer belimumab using the autoinjector, both at home and in the clinic. A small number of patients had assistance during their first injection in the clinic or required a second attempt. This is not unexpected in patients who may be self-administering an injection for the first time, as initial anxiety with self-injection has been reported previously in patients with RA [19]. In clinical practice, patients would likely receive support from their healthcare practitioner for first administrations, and therefore the study represents real-world clinical practice. 

With the targeted IV-to-SC switching interval of 1 to 4 weeks, patients’ belimumab concentrations reached steady-state concentration by week 2. For patients with a switch interval of more than 2.5 weeks between doses, the week-8 to week-1 difference in trough concentrations was close to zero. Patients with a switch interval of ≤ 2.5 weeks had a week-1 belimumab concentration higher than their week-8 steady-state concentration. This is not a safety concern as the median concentration before the first SC dose in the present study, where a minimum switching interval of 1 week was imposed, was substantially lower than the maximal belimumab concentration that is typically observed immediately after the last IV dose (313 µg/mL) [5]. Therefore, the PK results from this study support a 1 – 4-week interval for switching between belimumab IV and belimumab SC. In addition, the stable transition from IV to SC dosing demonstrated here supports the use of the prefilled syringe for administration of belimumab SC as bioequivalent exposures have been demonstrated for belimumab SC administration using the autoinjector and the prefilled syringe [15]. The 200-mg dose was selected based on previous analyses that showed that weekly belimumab 200 mg SC would achieve similar steady-state belimumab exposures to that of monthly belimumab 10 mg/kg IV [16]. The present results support this dose selection; the median concentration of 113 µg/mL is consistent with the simulated steady-state concentrations for weekly belimumab 200 mg (104 µg/mL) and monthly belimumab 10 mg/kg IV (110 µg/mL) [20]. Weekly SC dosing may be advantageous in terms of PK as fluctuations in steady-state concentrations are reduced compared with monthly IV administration due to more frequent dosing and slow SC absorption [20]. 

Patients with active SLE demonstrated a good level of usability and reliability with the autoinjector, confirming results of a previous study conducted in healthy subjects [15]. The majority of injection failures was due to user error, which were rectified with additional training and slight adjustments in user instructions. It was important that patients were comfortable and confident with the device, and when choosing between an SC or IV regimen (assuming comparable efficacy and safety profiles), patient preference and pharmacoeconomics should be taken into account [21]. The availability of an alternative formulation to belimumab IV will give patients the option to self-administer at home, avoiding the need to attend the infusion clinic every month. SC administration may have additional advantages, such as improved accessibility for patients with poor IV access and increased compliance and reduced costs, when clinic costs, travel expenses, and missing days from work or childcare are considered. In a claims database study in RA, patients who initiated treatment with SC biologics were significantly more likely to meet the criteria for high adherence compared with those who initiated treatment with IV biologics [22]. Costs were also lower for SC biologics, although it should be noted that these were not direct comparisons using the same biologic. Such comparisons of adherence and costs are not currently available in SLE. 

The safety data reported here are consistent with the known safety profile of belimumab IV, and there was a low incidence of SAEs. These safety data from 95 patients also support a switch from IV to SC administration. Injection-site pain must be considered when using SC medications, as this affects the patient’s overall experience [23] and willingness to continue. Here, in patients with SLE, pain levels were low, short-lived, and declined with repeated administration, as patients gained experience with the device and procedure. 

Our study objectives and patient population warranted an open-label design, which, along with the relatively short treatment duration, may be considered a limitation. Similarly, there may be some variability across the population and in the self-reported results from patient diaries, which assessed home use. However, the good level of usability and reliability demonstrated, directly assessed by the clinic site staff, is consistent with the self-reported home data. Efficacy was not examined, and biomarker data were not collected; efficacy of belimumab SC will be reported in a phase-3 randomized trial [24]. 

In conclusion, a novel autoinjector device had a good level of reliability and usability for self-administration of belimumab SC in patients with SLE and was well tolerated. PK data strongly support a time interval of 1 – 4 weeks, when switching between the IV and SC administration. 

## Acknowledgments 

Medical writing assistance was provided by Louisa Pettinger, PhD, of Fishawack Indicia Ltd, and was funded by GSK. 

## Conflict of interest 

This study was funded by GSK. SZS was an investigator in this study. NLF, JG, AEH, DR, and DG are full-time employees of GSK and hold stock options. HS is an employee of PAREXEL International contracted to GSK and holds stock in GSK. 

**Table 1.** Summary of administration errors.

	Wk 1 (n = 96)^a^	Wk 2 (n = 90)^a^	Wk 3 (n = 94)^a^	Wk 4 (n = 88)^a^	Wk 5 (n = 91)^a^	Wk 6 (n = 93)^a^	Wk 7 (n = 92)^a^	Wk 8 (n = 92)^a^
Use errors, n (%)								
Any use error	1 (1)	1 (1)	2 (2)	1 (1)	1 (1)	3 (3)	4 (4)	1 (1)
Autoinjector not properly activated on injection site	1 (1)^b^	0	0	0	0	0	0	0
Autoinjector pulled away before end of injection	0	1 (1)	1 (1)^b^	0	1 (1)	1 (1)	1 (1)^b^	1 (1)
Medication observed at the injection site	0	0	1 (1)	1 (1)	0	2 (2)	3 (3)	0
Device error, n (%)								
Any device error	0	0	0	0	1 (1)	1 (1)	2 (2)	0
Delivery stopped before end of injection	0	0	0	0	1 (1)^b^	0	0	0
Autoinjector did not activate	0	0	0	0	0	0	1 (1)^b^	0
Autoinjector leaked	0	0	0	0	0	1 (1)^c^	1 (1)^c^	0

^a^Number of injection attempts. In 5 cases, a patient had 2 attempts to administer their dose; ^b^the first attempt at injection was unsuccessful, the second attempt was successful; ^c^1 patient reported unsuccessful injections at week 6 and 7, which were initially attributed to both use error and device error; further investigation determined that the injections were unsuccessful due to use error only.

**Table 2. Table2:** Summary of adverse events.

	AEs, n	Patients, n (%) N = 95
Any AE^a^	105	39 (41)
Infections and infestations	27	18 (19)
Musculoskeletal and connective tissue disorders	20	12 (13)
General disorders and administration-site conditions	17	10 (11)
Drug-related AE^b^	25	15 (16)
General disorders and administration site conditions	10	6 (6)
Infections and infestations	9	6 (6)
Serious AE	7	4 (4)
Severe AE	2	2 (2)
AEs leading to discontinuation	5	3 (3)
Fatal AE	0	0

^a^Those occurring in > 10% are listed; ^b^those occurring in > 5% are listed (listed as system organ class). AE = adverse event; SAE = serious adverse event.

**Table 3. Table3:** Injection site pain.

Week	Time point	Mean VAS score (0 – 100 mm scale)	Acceptable, n (%)	
			Yes	No
Week 1	Dose	7.93	61 (65)	1 (1)
	1 hour	0.40	10 (11)	1 (1)
	24 hours	0.06	4 (5)	0
Week 2	Dose	8.15	44 (51)	2 (2)
	1 hour	0.74	9 (11)	0
	24 hours	0.36	3 (4)	0
Week 3	Dose	6.46	50 (56)	2 (2)
	1 hour	0.51	9 (10)	0
	24 hours	0.06	2 (2)	0
Week 4	Dose	4.08	35 (41)	1 (1)
	1 hour	0.21	4 (5)	0
	24 hours	0.01	1 (1)	0
Week 5	Dose	4.38	37 (41)	2 (2)
	1 hour	1.08	7 (8)	0
	24 hours	0.03	3 (3)	0
Week 6	Dose	3.28	35 (38)	3 (3)
	1 hour	0.12	4 (4)	0
	24 hours	0.02	2 (2)	0
Week 7	Dose	3.67	36 (40)	1 (1)
	1 hour	0.20	4 (4)	0
	24 hours	0.11	2 (2)	0
Week 8	Dose	2.30	28 (31)	1 (1)
	1 hour	0.31	2 (2)	0
	24 hours	0.18	2 (2)	0

VAS = visual analog scale.
